# Pan-cancer analysis reveals NUP37 as a prognostic biomarker correlated with the immunosuppressive microenvironment in glioma

**DOI:** 10.18632/aging.203862

**Published:** 2022-01-30

**Authors:** Ya He, Jingang Li, Lan Shen, Hui Zhou, Wei Fei, Guangliang Zhang, Zhen Li, Fei Wang, Yuetao Wen

**Affiliations:** 1 Department of Physical Examination Center, Jiangjin Central Hospital of Chongqing, Jiangjin, Chongqing 402260, China; 2 Department of Neurosurgery, Jiangjin Central Hospital of Chongqing, Jiangjin, Chongqing 402260, China; 3 Department of Neurology, Jiangjin Central Hospital of Chongqing, Jiangjin, Chongqing 402260, China

**Keywords:** NUP37, glioma, pan-cancer, tumor associated macrophages, immunosuppressive microenvironment

## Abstract

Nucleoporin 37 kDa (NUP37), a member of the nucleoporin family, has been reported to regulate the proliferation and apoptosis of several tumor types. However, its role in the tumor immune microenvironment is unclear. Here, we evaluated the expression, methylation, copy number alteration, and prognostic significance of NUP37 using RNA-seq and clinical data from The Cancer Genome Atlas. We observed higher expression of NUP37 in 28 of 29 tumor types, and high NUP37 expression predicted worse survival status of patients in 15 tumors. Using data from the cBioportal database, we described the gene variation of NUP37 in glioma and pan-cancer. We further assessed the role of NUP37 in the tumor immune microenvironment using immune infiltration data. NUP37 expression was positively associated with the infiltration levels of immunosuppressive cells, such as nTregs, iTregs, and tumor-associated macrophages, and negatively correlated with immune killer cells, such as CD8+ T and NK cells across cancers. Furthermore, NUP37 expression was associated with immune checkpoints and immune regulation-related genes. The half-maximal inhibitory concentrations of anti-cancer drugs were obtained from the Genomics of Drug Sensitivity in the Cancer database. The correlation between half-maximal inhibitory concentration and NUP37 expression was evaluated. The patients with the evaluated expression of NUP37 were resistant to several anti-cancer drugs. These results suggest that NUP37 is a potential oncogene and prognostic biomarker in glioma and pan-cancer. Tumor tissues with high NUP37 expression exist in a relatively immunosuppressive microenvironment and are resistant to several anti-cancer drugs.

## INTRODUCTION

Glioma is a kind of intracranial malignancies, which has very high recurrence rates and mortality. The World Health Organization (WHO) classifies gliomas into several grades. Grades II and III were defined as low-grade glioma (LGG), and grade IV was defined as glioblastoma multiforme (GBM) by WHO [1]. LGG may evolve into GBM with an average total survival of about 14.6 months [2]. Thus, only by identifying new biomarkers can we explore an effective treatment plan.

By now cancer treatment technology has been greatly improved. The progress of medical technology, especially the combination of different therapies, has improved the survival status of cancer patients [3, 4]. Immunotherapy, a revolutionary method of tumor therapy, has been applied in the clinical treatment of a variety of tumors [5]. However, immunotherapy, especially immune checkpoint inhibitors (ICIs), is only effective in a small number of patients. Thus, immunotherapy requires more biomarkers to predict efficacy in advance [6]. At present, most studies show that the effect of cancer treatment is related to tumor microenvironment, while relatively speaking, tumor immune microenvironment (TIME) is more important [7–9]. Tumor-associated macrophages (TAMs) and Tregs have a positive impact on immunosuppression and tumor [10]. The high infiltration level of TAMs and Tregs might predict a favorable response to ICIs treatment in many tumors [11], including brain tumors [12], and lung cancers [13], to name a few.

Nucleoporin 37 kDa (NUP37) belongs to the NUP family. It has been said NUP37 can regulate the growth and death of cancer cells [14, 15]. For example, knockdown of NUP37 could inhibit the proliferation of non-small lung cancer cells [16]. However, the immunomodulatory function and biomarker role of NUP37 in glioma and pan-cancer remain unclear.

In this study, universal cancer data were used to analyze the conveying, prognostic value, DNA methylation, copy number change (CNA) and mutation conditions about NUP37, and then the relationship with NUP37 conveying and the infiltration standard to immune cells and immune regulation-related genes was assessed. To find an ICI therapy more suitable for patients with NUP37 expression level.

## RESULTS

### Pan-cancer conveying of NUP37

**Figure 1:**
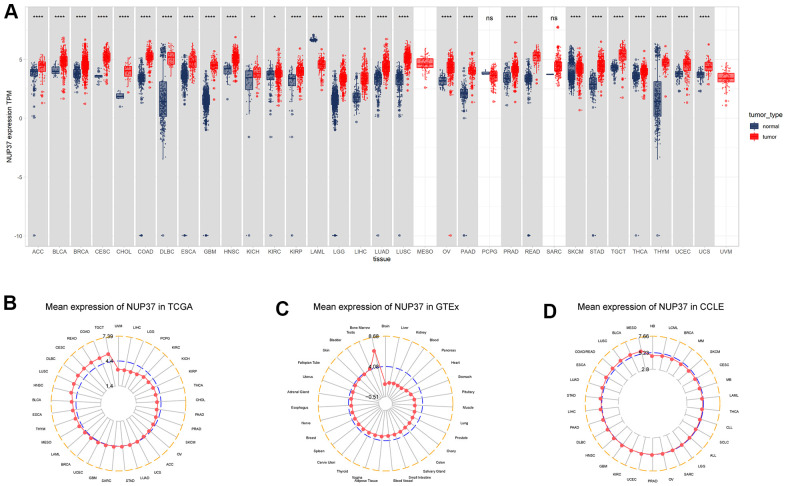
**Expression of NUP37.** (**A**) pan-cancer expression of NUP37. (**B**) NUP37 expression in tumor tissues from TCGA cohort. (**C**) NUP37 expression in normal tissues from GTEx cohort. (**D**) NUP37 expression in cancer cell lines from CCLE cohort. *P < 0.05, **P < 0.01, ****P < 0.0001.

First, the research explored the conveying of NUP37 about 31 tumor types with corresponding inform tissue employing the Cancer Genome Atlas and Genotype-Tissue Expression data. The results revealed that NUP37 was over-expressed by 28 of 29 tumor, it includes ACC, BLCA, BRCA, CHOL, COAD, DLBC, ESCA, GBM, HNSC, KICH, KIRC, KIRP, LGG, LIHC, LUAD, LUSC, OV, PAAD, PRAD, READ, SKCM, STAD, TGCT, THCA, THYM, UCEC, and UCS, but was lowly conveying in LAML (Figure 1A).

On the research we evaluated and assessed NUP37 conveying in tumor tissues of the TCGA cohort and considered NUP37 was most greatly expressed in TGCT and lowly expressed in UVM. The results in normal tissues of GTEx database showed NUP37 had the greatest conveying in bone marrow and the smallest expression in brain. In the study of tumor cell lines, it is necessary to use the data of the Cancer Cell Encyclopedia (CCLE) database to study, and it is found that NUP37 has the highest expression in MESO cell line.

**Figure 2:**
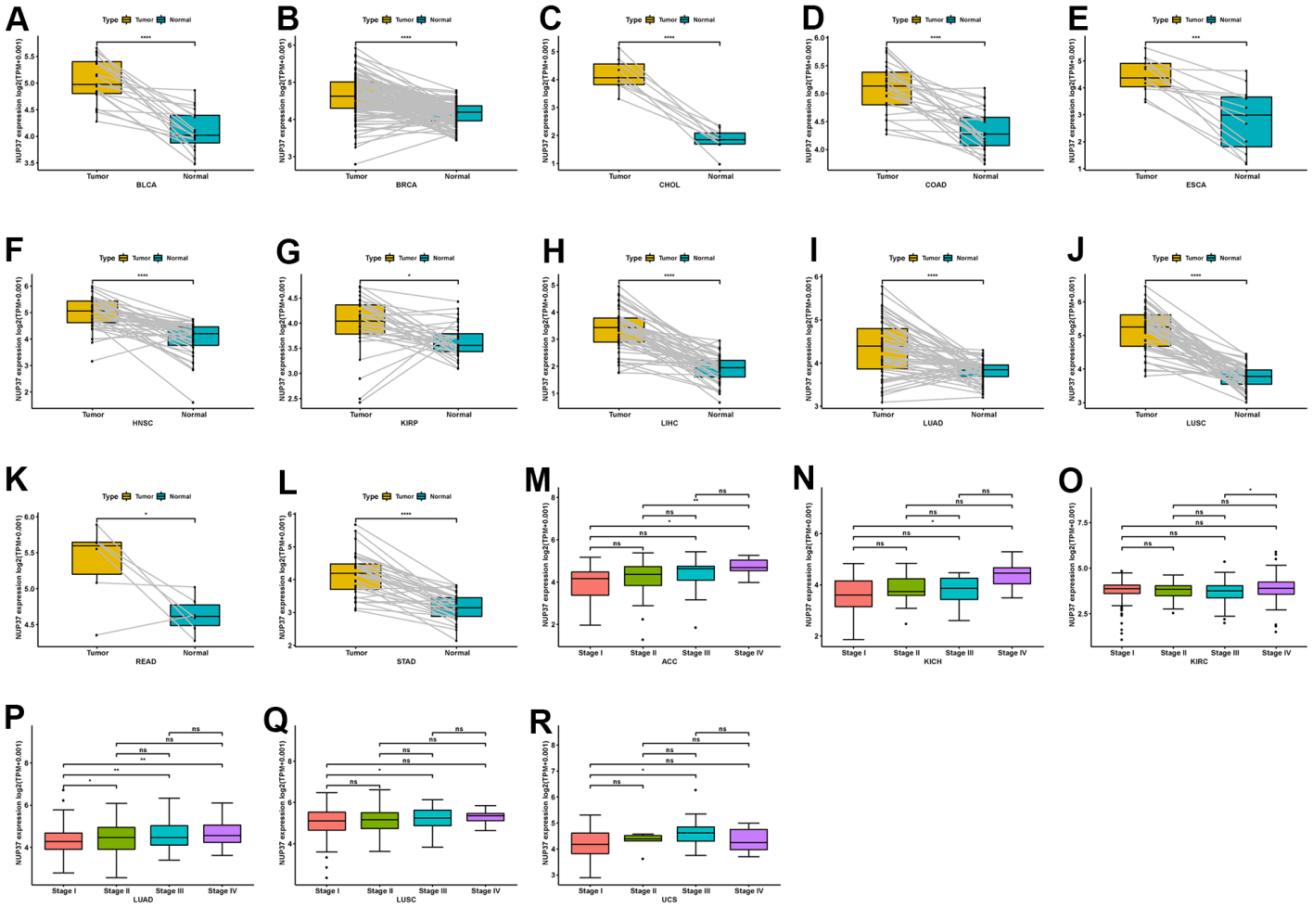
**Expression of NUP37 in paired tumor and adjacent normal tissues and various tumor stages.** (**A**–**L**) NUP37 expression in paired tumor and adjacent normal tissues from TCGA in indicated tumor types. (**M**–**R**) NUP37 expression in various tumor stages in indicated tumor types. *P < 0.05, **P < 0.01, ***P < 0.001, ****P < 0.0001.

Further, it assessed the conveying of NUP37 in couple tumors, tissues and at different tumor levels, and found that NUP37 was expressed in BLCA tissues, BRCA, CHOL, COAD, ESCA, HNSC, FLEA, LIHRP, RABD, LUS, LITERATURE and STAD (Figure 2A–2L). Then, in ACC, KIRC, RABRC, LUAD, LUSC and UCS, NUP37 were higher in the initial stage of tumor (Figure 2M–2R).

**Figure 3:**
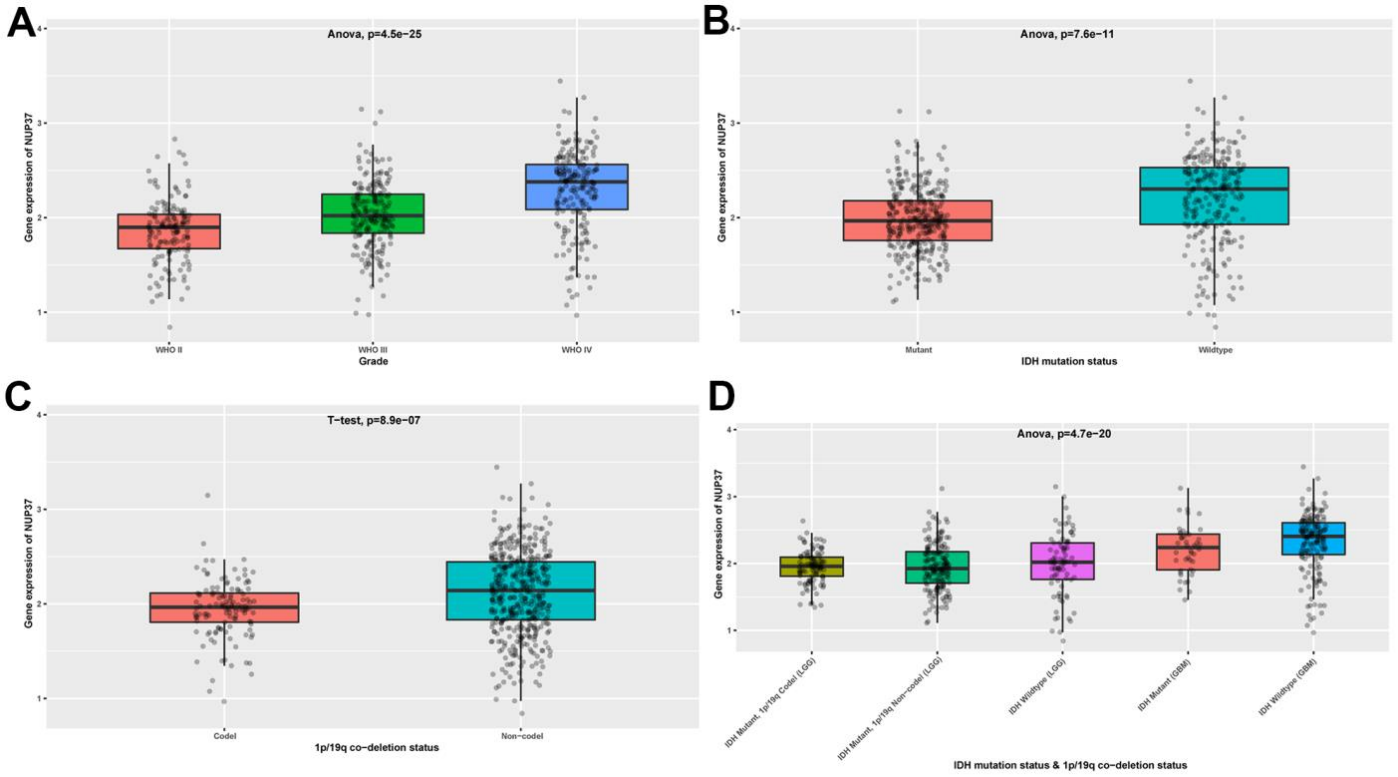
**Expression of NUP37 in glioma.** (**A**) NUP37 expression in various tumor stages of glioma patients from CGGA cohort. (**B**) NUP37 expression in indicated IDH mutation status of glioma patients from CGGA cohort. (**C**) NUP37 expression in indicated 1p/19q co-deletion status of glioma patients from CGGA cohort. (**D**) NUP37 expression in indicated groups of glioma patients from CGGA cohort.

Third, in the Chinese Glioma Genome Atlas (CGGA) database, we found that NUP37 expression was higher in later tumor stages of glioma (Figure 3A). Glioma patients with wild-type IDH mutation status or non-codeletion of 1p/19q status have a worse survival status [17]. We observed that NUP37 expression was higher in wild-type IDH mutation status (Figure 3B) and non-codeletion of 1p/19q status (Figure 3C). In addition, NUP37 expression was the highest in patients with wild-type IDH mutation status (Figure 3D).

### Gene alteration of NUP37

**Figure 4:**
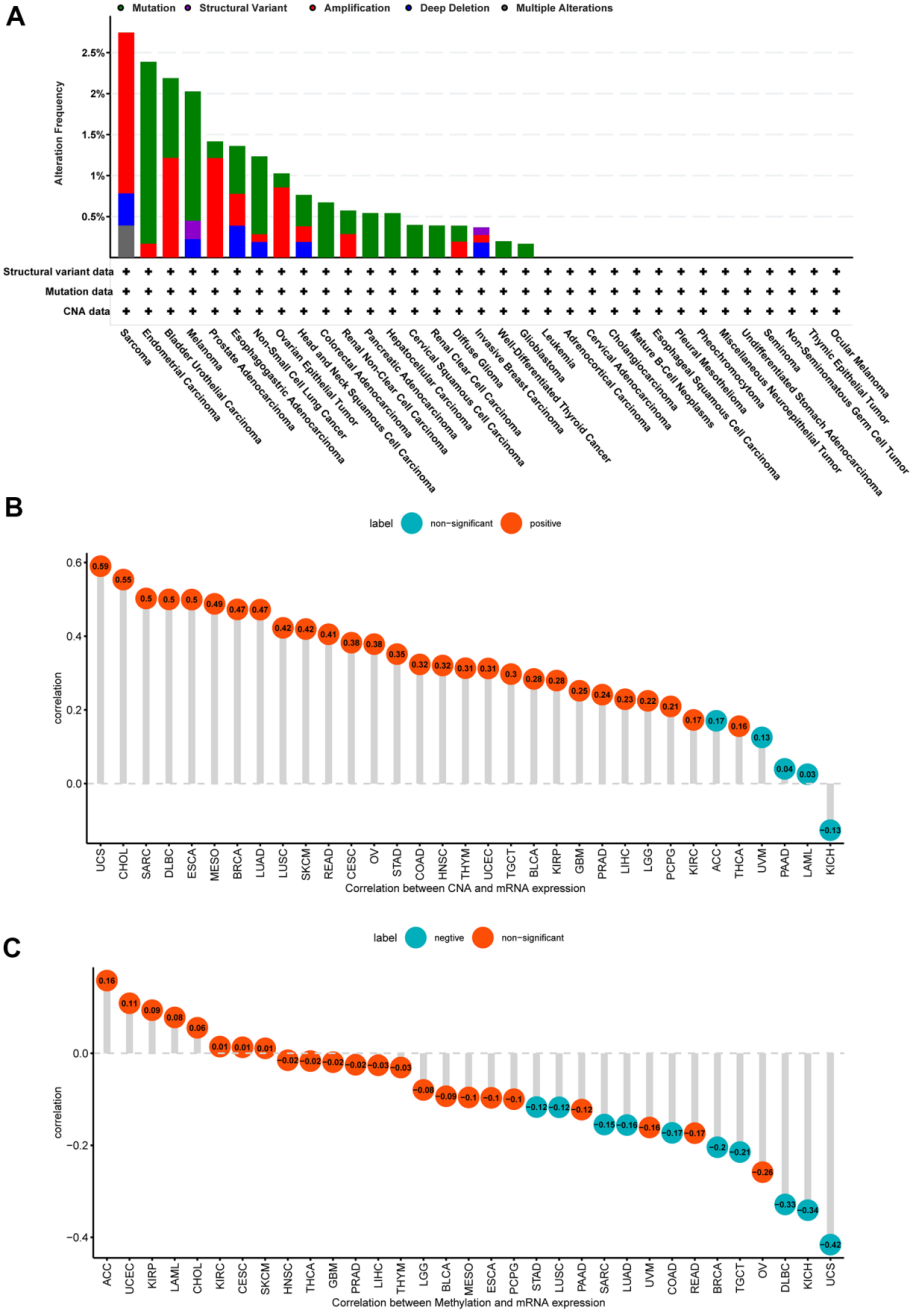
**Gene alteration of NUP37.** (**A**) The gene alteration of NUP37 in TCGA pan-cancer using cBioportal database. (**B**) The correlation between NUP37 expression and CNA. (**C**) The correlation between NUP37 expression and DNA methylation.

In addition, we assessed the NUP37 mutation, CNA and methylation conditions in the cancer pan. The study believed that the nup337 genomic modification was higher than 2.5% in SARC patients, of which the first type was “Amplification” (Figure 4A). For the nup37 and CNA correlation, it discovered NUP37 conveying was closely associated within CNA in GBM (r=0.25, p<0.05), LGG (r=22, p<0.05) and other types of data (Figure 4B). The study shows go a step the active methylation standard of NUP37 showed no correlation with NUP37 conveying in GBM and LGG (Figure 4C). These consequences show that the CNA conditions take effect on NUP37 in high expression.

### Significance of NUP37 expression

**Figure 5:**
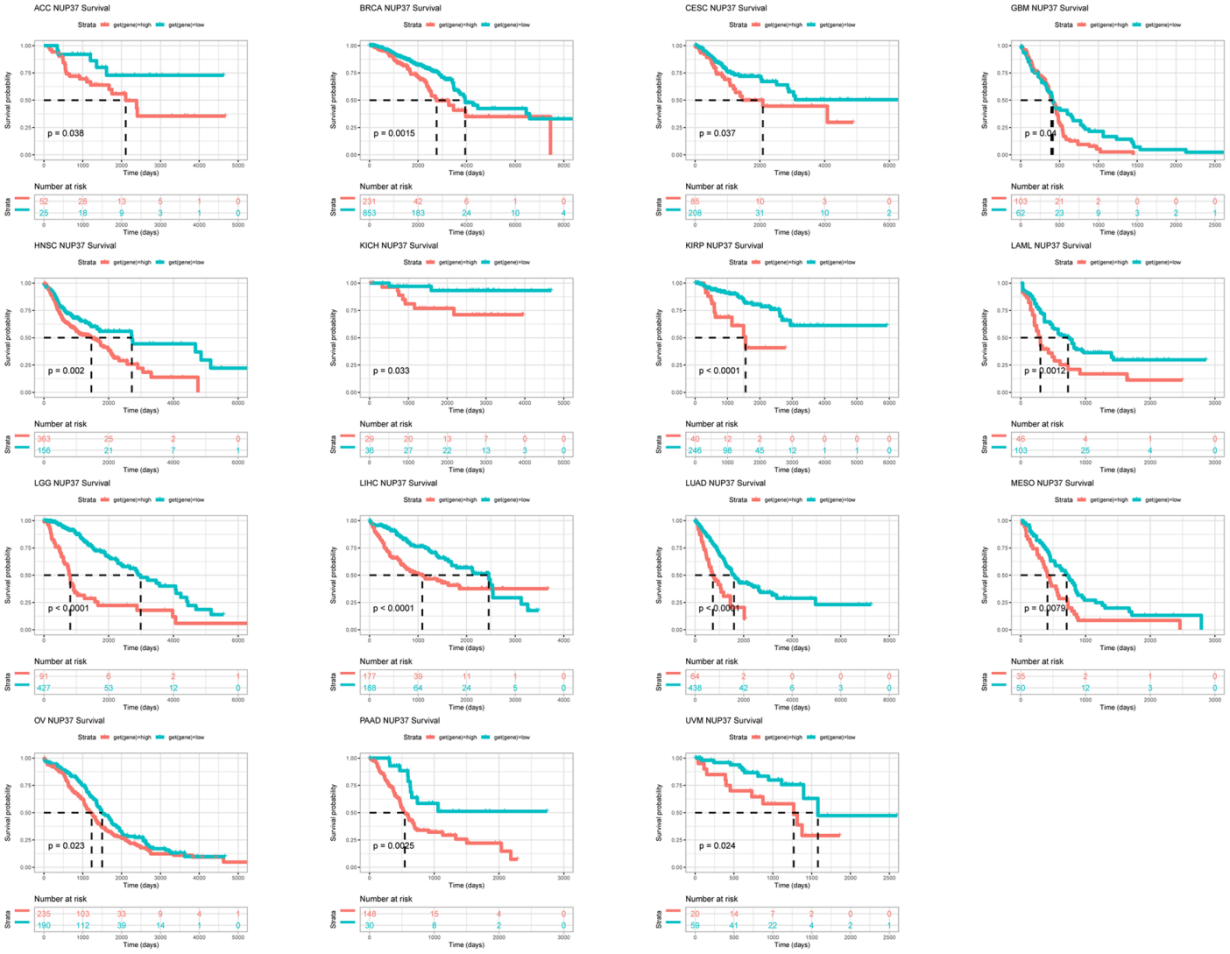
**Prognostic significance of NUP37 for OS of patients.** Kaplan-Meier OS results of NUP37 in pan-cancer. The best cut-off of NUP37 expression was set as cut-off value.

**Figure 6:**
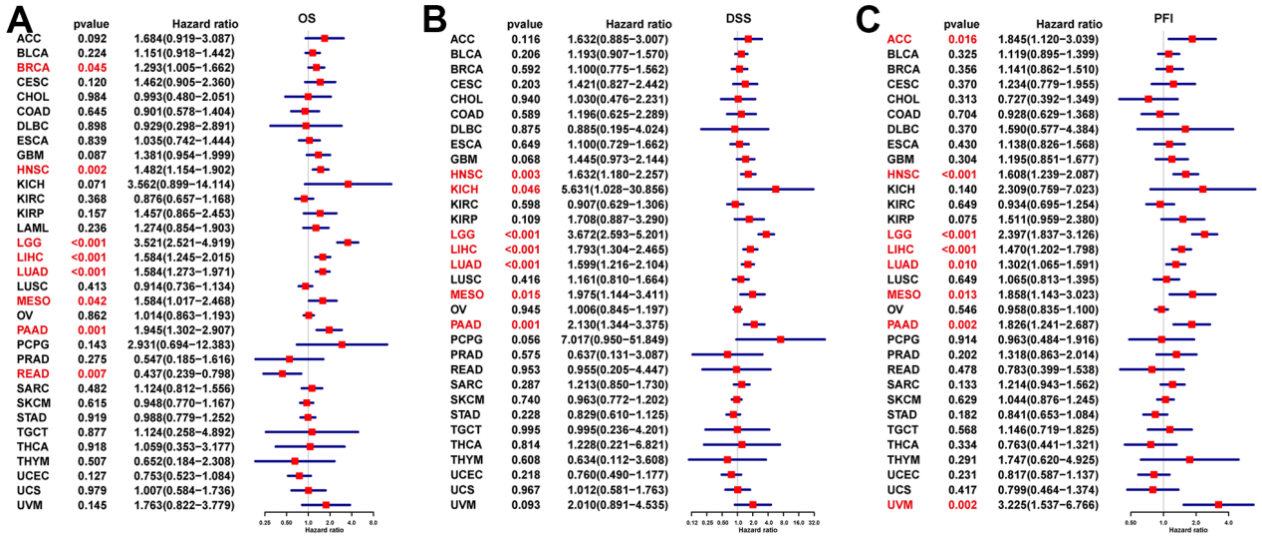
**Prognostic significance of NUP37 for OS, DSS, and PFI of patients.** The uniCox results of NUP37 in pan-cancer for OS (**A**), DSS (**B**), and PFI (**C**) of patients. Red color represents significant results (p < 0.05).

We further performed Kaplan–Meier analysis and univariate Cox regression (uniCox) analysis to evaluate the influence of NUP37 on the survival status of patients. In Kaplan-Meier analysis of OS, it was found that a high conveying of NUP37 with 15 of 33 neoplasm of TCGA cohort was related to the worse OS of patients, it may be have ACC, BRCA, CESC, GBM, HNSC, KICH, KIRP, LAML, LGG, LIHC, LUAD, MESO, OV, PAAD and UVM (Figure 5). Furthermore, the results of the uniCox OS showed NUP37 was a hazard for BRCA, HNSC, LGG, LIHC, LUAD, MESO, and PAAD, by contrast a protective factor in READ (Figure 6A). For disease-specific survival (DSS) results, NUP37 was a hazard view in HNSC, KICH, LGG, LIHC, LUAD, MESO, and PAAD (Figure 6B). For progression-free interval (PFI), which is a major NUP37 conveying predicted shorter PFI times in invalids who have ACC, HNSC, LGG, LIHC, LUAD, MESO, PAAD (Figure 6C).

### Gene set enrichment analysis (GSEA) of NUP37

**Figure 7:**
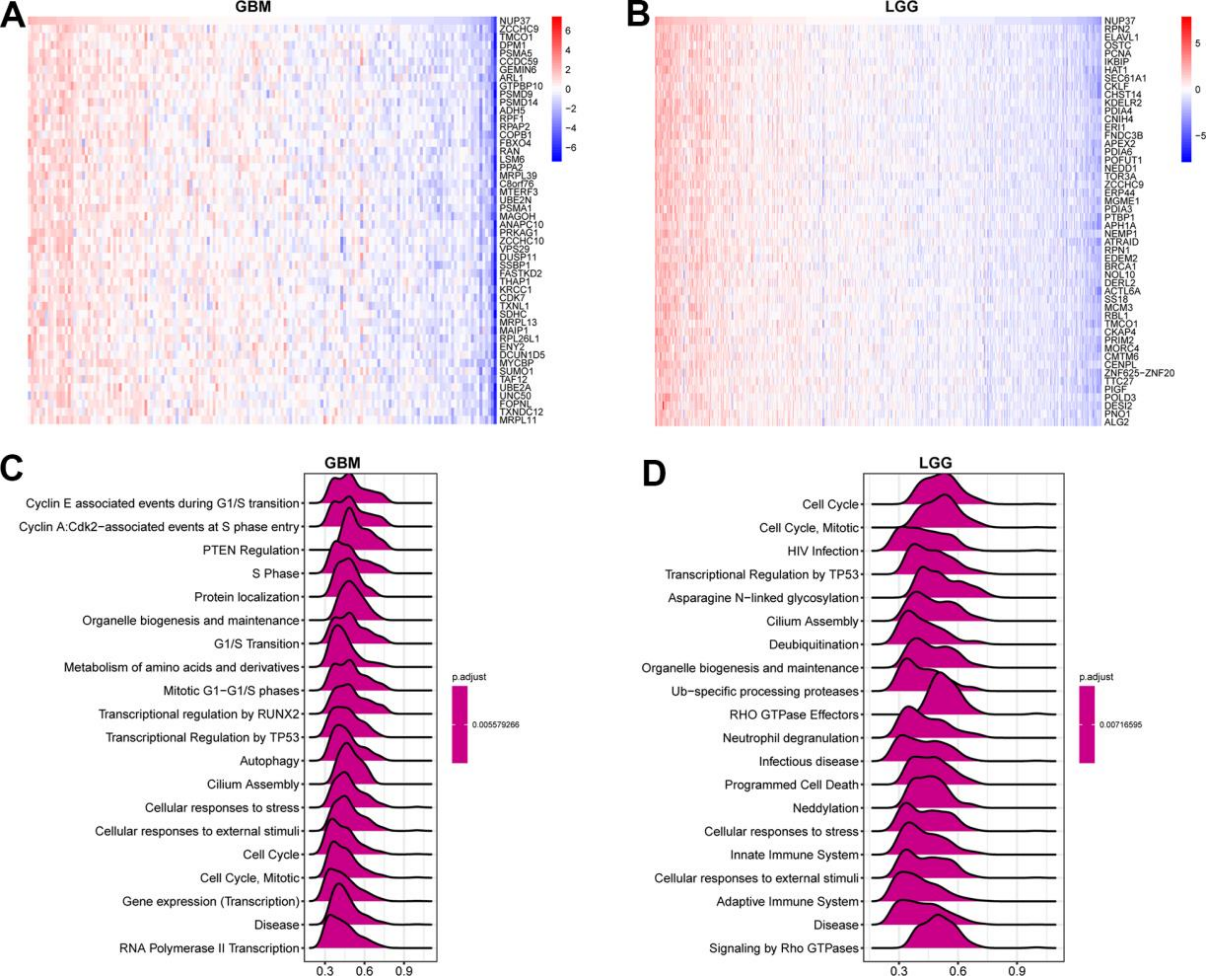
**GSEA of NUP37.** (**A**) The expression of top 50 genes correlated with NUP37 expression in GBM. (**B**) The expression of top 50 genes correlated with NUP37 expression in LGG. (**C**) The top 20 GSEA-Reactome results were showed in GBM. (**D**) The top 20 GSEA-Reactome results were showed in LGG.

Through this study, GSEA can predict its contribution to NUP37. The correlation analysis of GSEA (Figure 7A, 7B variety) was carried out and the gene nup37 (p<0.05) was sequenced. We used the R package “clusterProfiler” to analyze the Reactome pathway (GSEA-Reactome) terms in GBM and LGG. The GSEA-Reactome results in GBM proved that NUP37 was enriched in the “Cell Cycle” and “Autophagy” pathways (Figure 7C). The results of GSEA-Reactome analysis in LGG showed that NUP37 was related to the cell cycle-and immune-regulation-related pathways, for instance cell cycle, adaptive immune system, and congenital immunity (Figure 7D). The final results show that the low survival rate of glioma patients may be caused by the cell cycle and immunomodulatory function of NUP37.

### Immune cell infiltration analysis of NUP37

**Figure 8:**
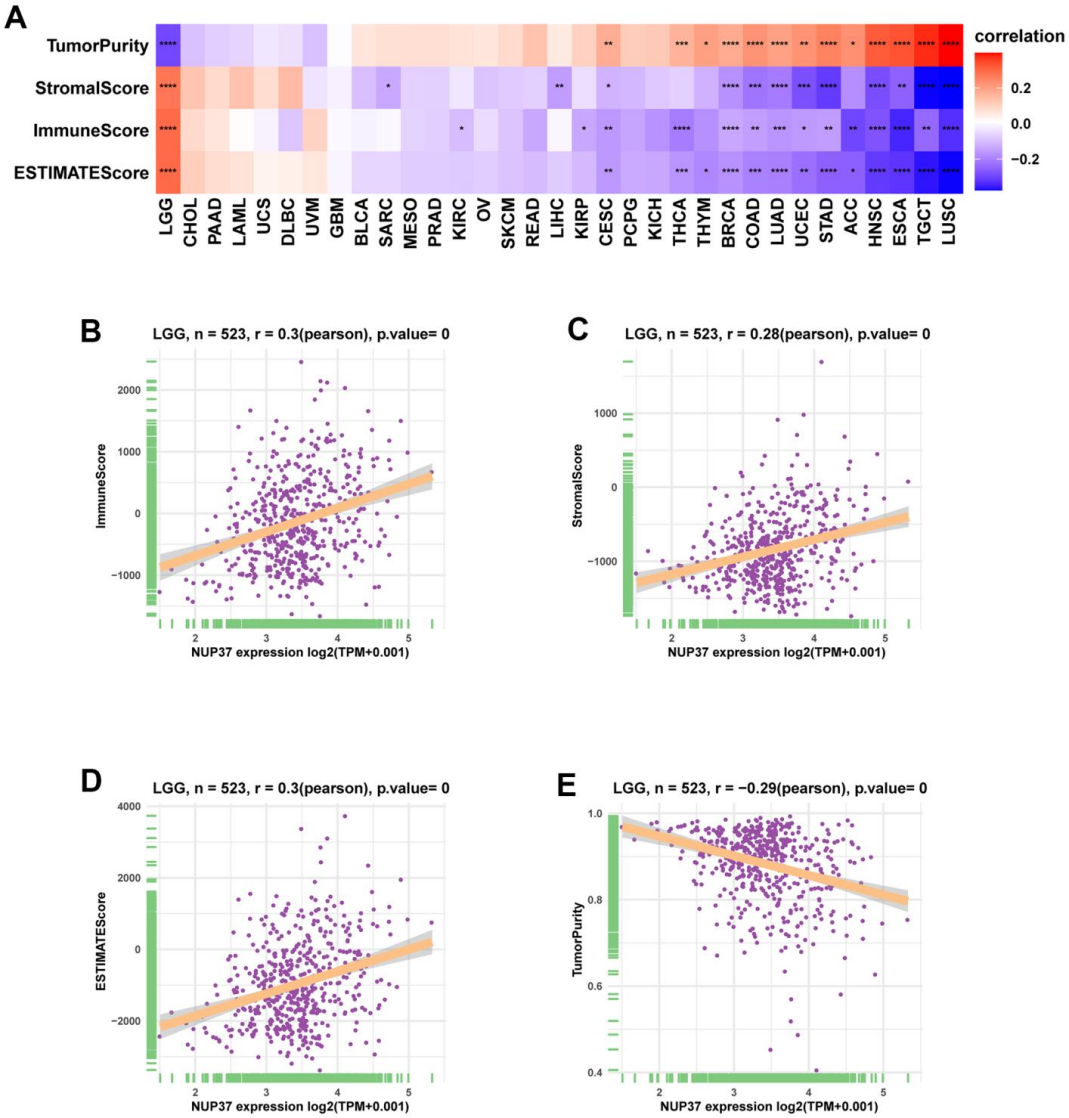
**Tumor microenvironment analysis of NUP37.** (**A**) Heatmap represents the correlation between NUP37 expression and TME scores in pan-cancer. (**B**–**E**) The correlation between NUP37 expression and immune score (**B**), stromal score (**C**), ESTIMATE score (**D**), and tumor purity score (**E**) in LGG. *P < 0.05, **P < 0.01, ***P < 0.001, ****P < 0.0001.

**Figure 9:**
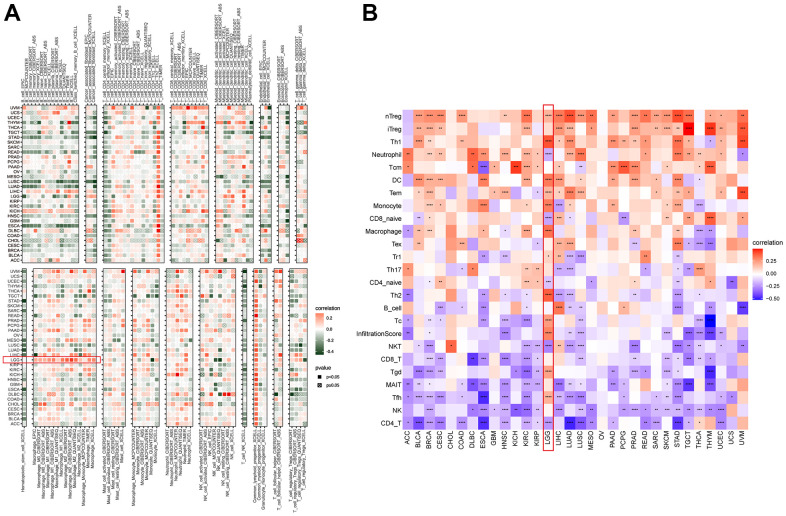
**Immune infiltration analysis.** (**A**) The correlation between NUP37 expression and infiltration levels of immune cells downloaded from TIMER2. Red represents positive correlation, green represents negative correlation, and the darker the color, the stronger the correlation. (**B**) The correlation between NUP37 expression and infiltration levels of immune cells downloaded from ImmuCellAI database. Red represents positive correlation, blue represents negative correlation, and the darker the color, the stronger the correlation. *P < 0.05, **P < 0.01, ***P < 0.001, ****P < 0.0001.

To understand the immune-regulation function of NUP37, the relationship between NUP37 conveying, stromal and immune scores was computed by R package “ESTIMATE” in pan-cancer (Figure 8A). The results showed that in LGG, NUP37 showed the same changes as immune score (Figure 8B), matrix score (Figure 8C) and estimated score (Figure 8D), but was opposite to tumor purity (Figure 8E). The correlation analysis of immune cell infiltration data downloaded from TIMER2 database showed that the expression of NUP37 in LGG was consistent with TAMs height (Figure 9A). We obtained data regarding 24 immune cells in the ImmuCellAI database. The analysis results such as nTregs, iTregs and TAMs of LGG (Figure 9B) show that the expression level of NUP37 is positively correlated with the level of immunosuppressive cells.

**Figure 10:**
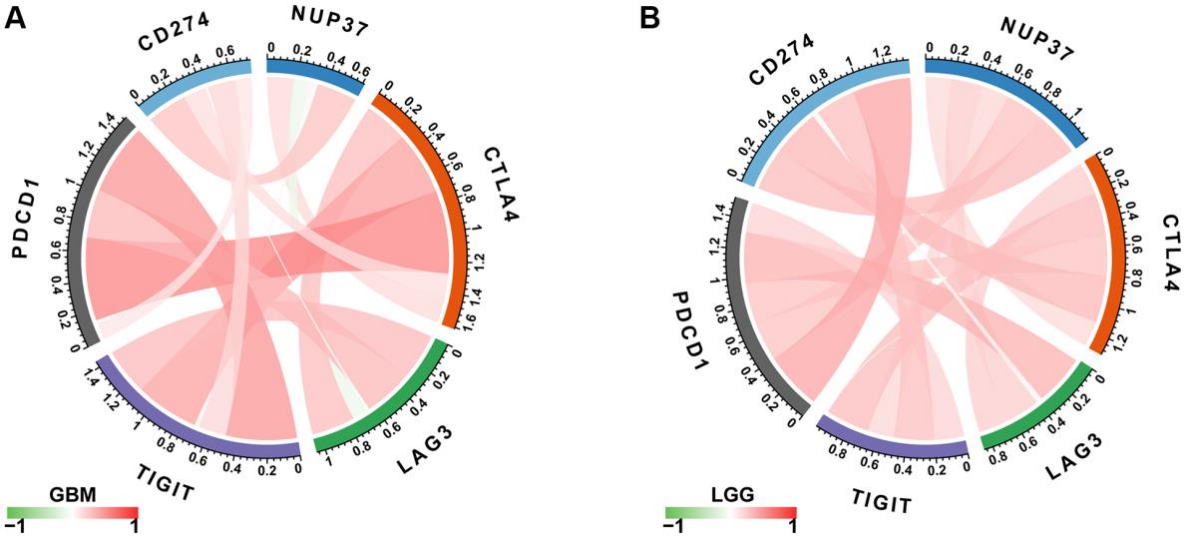
**Correlation between immune checkpoints.** (**A**) The correlation between NUP37 expression and immune checkpoints in GBM. (**B**) The correlation between NUP37 expression and immune checkpoints in LGG. Red lines represent positive correlation, green lines represent negative correlation, and the darker the color, the stronger the correlation.

**Figure 11:**
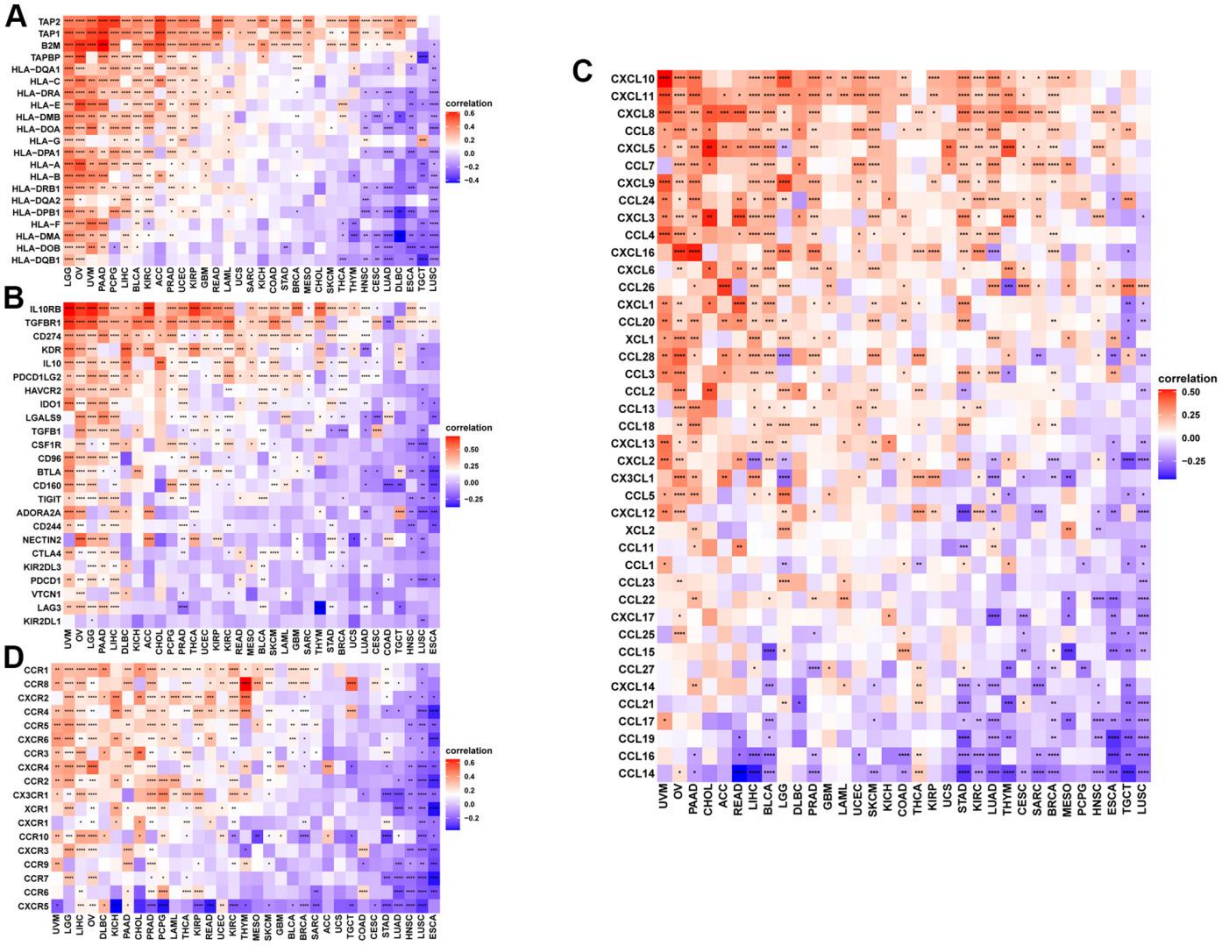
**Correlation between immune regulation related genes.** (**A**–**D**) The correlation between NUP37 expression and MHC genes (**A**), immunosuppressive genes (**B**), chemokines (**C**), and chemokine receptors (**D**). *P < 0.05, **P < 0.01, ***P < 0.001, ****P < 0.0001.

The study further showed that the expression of NUP37 has a positive correlation with immune checkpoints in GBM (Figure 10A) and LGG (Figure 10B), such as CD274, PDCD1, TIGIT, LAG3 and CTLA4. At the same time, the overcomes of this study reveal that patients who have high NUP37 expression may have a relatively immunosuppressive environment. We then explored the relationship in NUP37 and immune-related genes. In most tumors, especially in LGG, NUP37 is closely related to MHC gene (Figure 11A), immunosuppressive gene (Figure 11B), chemokine (Figure 11C) and chemokine receptor (Figure 11D) in most tumors, especially in LGG.

### Analysis of tolerance

**Figure 12:**
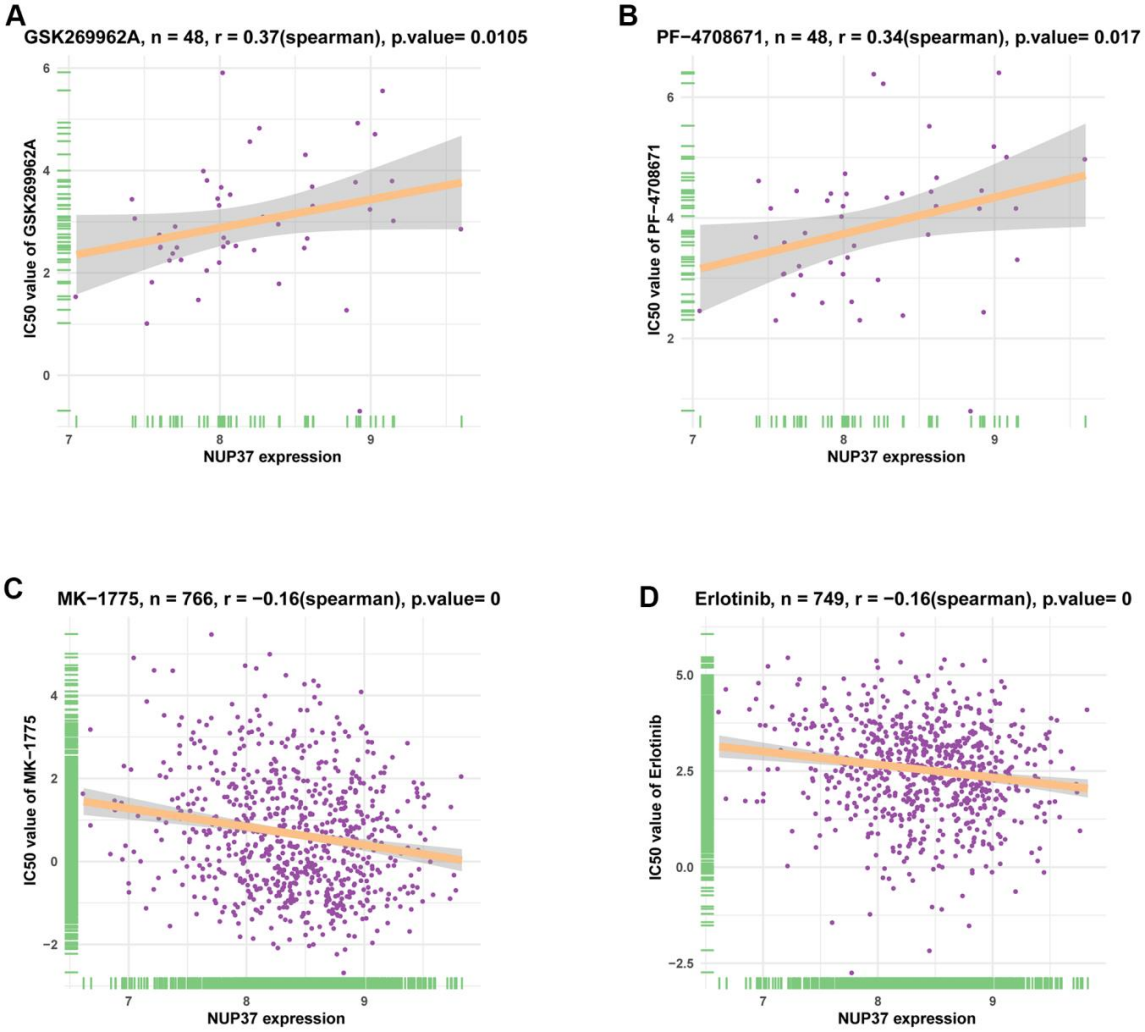
**The correlation between NUP37 expression and IC50 values of anti-cancer drugs.** (**A**–**D**) The correlation between NUP37 expression and IC50 values of indicated anti-cancer drugs.

At last, we analyzed the correlation between NUP37 and half-maximal inhibitory concentration (IC50) of 192 anti-cancer drugs using the Genomics of Drug Sensitivity in Cancer (GDSC) database. The results showed that NUP37 are positively related to IC50 between the two anticancer drugs PF-470871 and GSK26962 (Figure 12A, 12B and Supplementary Table 1). In contrast, the NUP377 expression is negatively correlated with IC50 of 57 anti-cancer drugs, such as MK-1775 and Erlotinib (Figure 12C, 12D and Supplementary Table 1).

## DISCUSSION

Although immunotherapy brings new hope to cancer patients, many patients remain insensitive to immunotherapy [18, 19]. Some relative researches have proved that the remodeling of TIME by cancer cells pay an emphasis on the resistance to immunotherapy, which to reduce the response of tumor patients to treatment and it may be result in worse survival status [20, 21]. Therefore, there is an urgent need to identify essential genes that may affect time.

NUP37 expression has been evaluated in most tumor types. In glioma, Liu et. indicated that high NUP37 expression is significantly correlated with reduced overall survival [14]. However, the immunomodulatory function and biomarker role of NUP37 in glioma and pan-cancer remains unclear. This study was the first to assess the expression of NUP37 pan-cancer. Our analysis found that NUP37 expression was highly expressed in 28 of 29 tumor types, including ACC, BLCA, BRCA, CHOL, COAD, DLBC, ESCA, GBM, HNSC, KICH, KIRC, KIRP, LGG, LIHC, LUAD, LUSC, OV, PAAD, PRAD, READ, SKCM, STAD, TGCT, THCA, THYM, UCEC, and UCS.

The significance of prognosis evaluation of NUP37 requires Kaplan-Meyer survival analysis using the data of TCGA database. Finally, Kaplan-Meyer OS analysis concluded that the increased expression of NUP37 indicated that the OS of patients with 15 tumor types was poor, including GBM and LGG. To explore the mechanism with this typical prognosis of NUP37, we conducted a GSEA study and believed that immunomodulatory pathways are ample in gliomas, which shows the important role of NUP37 in real TIME. We strongly affirmed the results to use the immune cells intrusion in the TIMER2 and ImmuCellAI databases. This analysis suggested that NUP37 was extremely suppress immune infiltration, including iTregs, nTregs, and TAMs in glioma and pan-cancer. In contrast, NUP37 was negatively correlated with immune killer cells, including NK cells and CD8+ T cells. Moreover, we also revealed that NUP37 expression was a positively correlation between immune checkpoints, immunosuppressive genes, and immune regulation-related genes of glioma and pan-cancer. These investigations confirm the result that patients who have high NUP37 conveying may have a extremely suppress immune circumstance. What is more, we analyzed the correlation between NUP37 and IC50 of 192 anticancer drugs. The results suggested that invalids who have high NUP37 expression might resist to some anti-cancer drugs, such as PF-4708671 and GSK269962A, at the same time have sensitive to most anti-cancer drugs, such as MK-1775 and erlotinib.

However, our study had several limitations. (1) Our study lacks in-depth experimental verification and mechanistic research on NUP37 in glioma. In future research, we will conduct in-depth research in this field. (2) We suspect that the high expression of NUP37 leads to an immunosuppressive microenvironment in glioma, resulting in immunotherapy tolerance. Knocking down the expression of NUP37 may help increase the sensitivity of immunotherapy. This aspect needs to be confirmed through *in vivo* experiments and in-depth clinical studies.

To be short, the research revealed that NUP37 is an oncogene and a prognostic marker in glioma and pan-cancer, which thinks high NUP37 expression may promote an immunosuppressive microenvironment.

## MATERIALS AND METHODS

### Datasource

The RNA-seq data and corresponding clinical data from the TCGA, GTEx, then downloading CCLE databases, glioma patients’ gene expression data and corresponding clinical data from the UCSC XENA website (https://xenabrowser.net/datapages/) and the CGGA dataset (http://www.cgga.org.cn/index.jsp). Then downloading from the UCSC XENA website gene mutation information and the methylation and copy number alteration information about NUP37 in cBioPortal database.

### Analysis of prognosis

The analysis of Kaplan–Meier and uniCox were ran in evaluate and use R package called “survminer” and “survival”. OOS, DSS, and PFI levels were levels were evaluated.

### Correlation analysis and GSEA

We find the pertinence in NUP37 and all protein-coded mRNAs in the pan-cancer samples in TCGA. The mRNAs correlated with NUP37 (Pearson’s correlation coefficient, p < 0.05) were ranked and subjected to GSEA using the R package “clusterProfiler”.

### Correlation between NUP37 level and immune cell infiltration

The stromal and immune scores of samples in TCGA pan-cancer were calculated employing the R package “ESTIMATE.” There are two ways to assess the relationship between the level of NUP37 and the level of the immune cell infiltration. In the first method, we obtained the infiltration level of immune cells from the TIMER2 database (http://timer.comp-genomics.org/). In the second method, we downloaded the infiltration data of 24 immune cells from the ImmuCellAI database (http://bioinfo.life.hust.edu.cn/ImmuCellAI#!/).

### Analysis of tolerance

The IC50, gene expression profiles and clinical information of 192 drugs and 809 cell lines were downloaded from GDSC (https://www.cancerrxgene.org/). The correlation between NUP37 expression and the IC50 values of 192 drugs was analyzed, and the Spearman’s correlation coefficients were calculated.

### Statistical analysis

In this study, R software (version 4.1.0) was used for statistical analysis. Including all statistical methods of the whole study and appropriate R. The distinctiveness is set as p < 0.05.

## Supplementary Material

Supplementary Table 1
